# Three-Dimensional CT-Based Limb Length Evaluation Is Highly Dependent on Anatomical Landmark Selection and Pelvic Asymmetry

**DOI:** 10.1016/j.artd.2023.101206

**Published:** 2023-09-18

**Authors:** William B. O’Callaghan, Matt Thompson, Thies Wuestemann, Sarah L. Whitehouse, Ross W. Crawford

**Affiliations:** a Department of Orthopaedics – Queensland Health, The Prince Charles Hospital, Chermside, Queensland, Australia; b Orthopaedic Research Institute of Queensland (ORIQL), Townsvillle, Queensland, Australia; c Stryker Corporation, Kalamazoo, MI, USA; d Queensland University of Technology, Kelvin Grove, Queensland, Australia

**Keywords:** Total hip arthroplasty, Pelvic asymmetry, Global offset, Hip length, Robotics, Digital templating

## Abstract

**Background:**

Pelvic skeletal asymmetry can result in rotational differences and morphologic bony prominence variance between the left and right hemipelvis. When selecting bony reference points for modern computed tomography-based robotic total hip arthroplasty planning, it is unclear which bony landmarks are the most reliable and accurate, especially in the presence of significant pelvic asymmetry.

**Methods:**

A retrospective study was conducted utilizing a database of computed tomography scans. Multiple bony landmarks in the pelvis and femur were selected for comparison, with the aim of measuring pelvic asymmetry. Specifically, the study measured the average difference in lateral offset between the left and right hemipelvis caused by pelvic asymmetry. Landmarks were also compared to determine the impact of pelvic asymmetry on hip length, femur length, and limb length discrepancies. Furthermore, a scenario was simulated in the software whereby a total hip replacement was inserted, potentially changing the hip length. The impact of pelvic reference point selection on the measurement of this simulated change in hip length was examined.

**Results:**

This study population showed widespread pelvic asymmetry. The anatomical landmarks of the opposite side cannot be relied upon for predicting the anatomy of the affected side. The center of rotation axis is more reliable than the inferior obturator foramen axis for hip length discrepancy due to pelvic asymmetry (*P* < .05).

**Conclusions:**

Current computer-assisted surgery THR software reports measurements of global offset and hip length that do not consider pelvic asymmetry. Surgeons are not given confidence ranges to represent the potential impact of asymmetry on the global offset and hip length values. Surgeons following these numbers to guide implant position may incur implant placement error should significant pelvic asymmetry be present in a given patient.

## Introduction

In the study of human osteology, numerous authors have examined the phenomenon of human skeletal asymmetry between the left and right (L/R) sides of the body for both the upper and lower appendicular skeleton. [[1], [2], [3], [4], [5], [6], [7]] Asymmetry of the axial skeleton including the pelvis has also been examined [8,9] with several authors noting that pelvis asymmetry is often associated with various internal and external pathological entities. [[10], [11], [12]] These pathologies vary from scoliosis to limb length discrepancies (LLDs) of different etiologies, neuromuscular pathologies, sacroiliac dysfunction, and trauma [[13], [14], [15], [16]]. Boulay et al.’s [17] anatomical study demonstrated that there are competing torsional forces acting on the pelvis in the axial plane that result in a unidirectional asymmetry of the pelvis osteology. This rotational asymmetry can compromise the limb length assessment when the anterior superior iliac spine (ASIS) is used as a landmark during clinical measurement as compared to a pubic tubercle reference point. Additionally, demographic variations exist in bony morphology of various patient populations. Badii et al. [9] examined pelvic computed tomography (CT) scans, defining pelvic asymmetry as the presence of an unequal distance from the iliac crest to the acetabuli, and demonstrated that 5.3% of the subjects had pelvic asymmetry >5 mm and 0.6% had pelvic asymmetry >10 mm.

Total hip arthroplasty has been shown to be a dependable procedure for pain relief. However, the restoration of constitutional anatomy including limb length and hip offset are also critical to restoring patient function and biomechanics. Limb length discrepancy (LLD) following total hip replacement is a common problem and a significant source of patient dissatisfaction [18]. Published radiographic and clinical measures of LLD can vary greatly in value depending on the method [19]. Although some studies have shown agreement between clinical tape measurement methods and CT scans from the ASIS to the medial malleolus (MM), only the “apparent” or “relative” method of LLD (umbilicus to L/R medial malleoli) has shown a significant difference between unperceived LLD and perceived LLD values [18,20]. For instance, only 36% of patients with a radiographically determined LLD (5 mm or greater) perceive a LLD [21]. Unlike radiographic and clinical measurements, image-based robotic and navigation total hip systems allow precise three-dimensional (3D) bony assessment of hip or limb length changes relative to the preoperative condition and, in some cases, relative to the contralateral limb (ie, discrepancy) [22].

Unfortunately, there is no standard or consensus as to which 3D bony landmarks on the pelvis or limb most accurately represent the clinical condition of pelvic asymmetry. This is relevant when considering which bony landmarks to use as reference points for CT-based navigation for robotic hip arthroplasty. The objective of this study is to investigate the influence of left/right pelvic asymmetry on pelvic offset and assess its impact on hip, femur, and limb length differences within the context of robotic hip arthroplasty navigation. Additionally, the study aims to identify the most accurate bony landmarks for determining differences in hip, femur, and limb length due to left/right pelvic asymmetry. Furthermore, the study seeks to evaluate the accuracy of different landmarks in detecting a simulated change in hip length.

## Material and methods

This study was a retrospective database analysis conducted using the proprietary software SOMA (Stryker Orthopaedic Modeling Analytics; Stryker, Mahwah, NJ), comparing the accuracy of different bony landmarks when measuring hip length, femoral length, and limb length due to pelvic asymmetry. Additionally, pelvic offset due to asymmetry and the effect of asymmetry on simulated length perturbations were measured.

SOMA is an analytic tool that contains an expansive database of femoral, pelvic, and tibial computed tomography scans acquired from patients across the globe: Germany/France/United States (∼78%), Japan/China/South Korea (∼16%), India/Middle Eastern/unlisted (∼5%) [23,24]. The software is able to allow users to create virtual constructs on a desired reference bone from predefined landmarks or user-defined points. The software then uses a series of transformations to map these constructs from the reference bone to every bone in the database, thus allowing the user to quantitatively evaluate the morphology of all bones in the database and apply various measurements between defined landmarks [23]. This allows the user to accurately, repeatedly, and efficiently assess bone morphology and to consistently identify landmarks on the bony anatomy within the large database. The bone morphology measurements can be analyzed within the software application as well as exported to a Microsoft Excel spreadsheet for postprocessing. Anonymized patient information such as ethnicity, gender, height, and weight are also included in the database. Ethical approval was obtained from each country where CT scans were acquired.

Only patients aged 50 years or older were selected from the SOMA database, as they are more likely to be candidates for total hip procedures.

**Figure 1:**
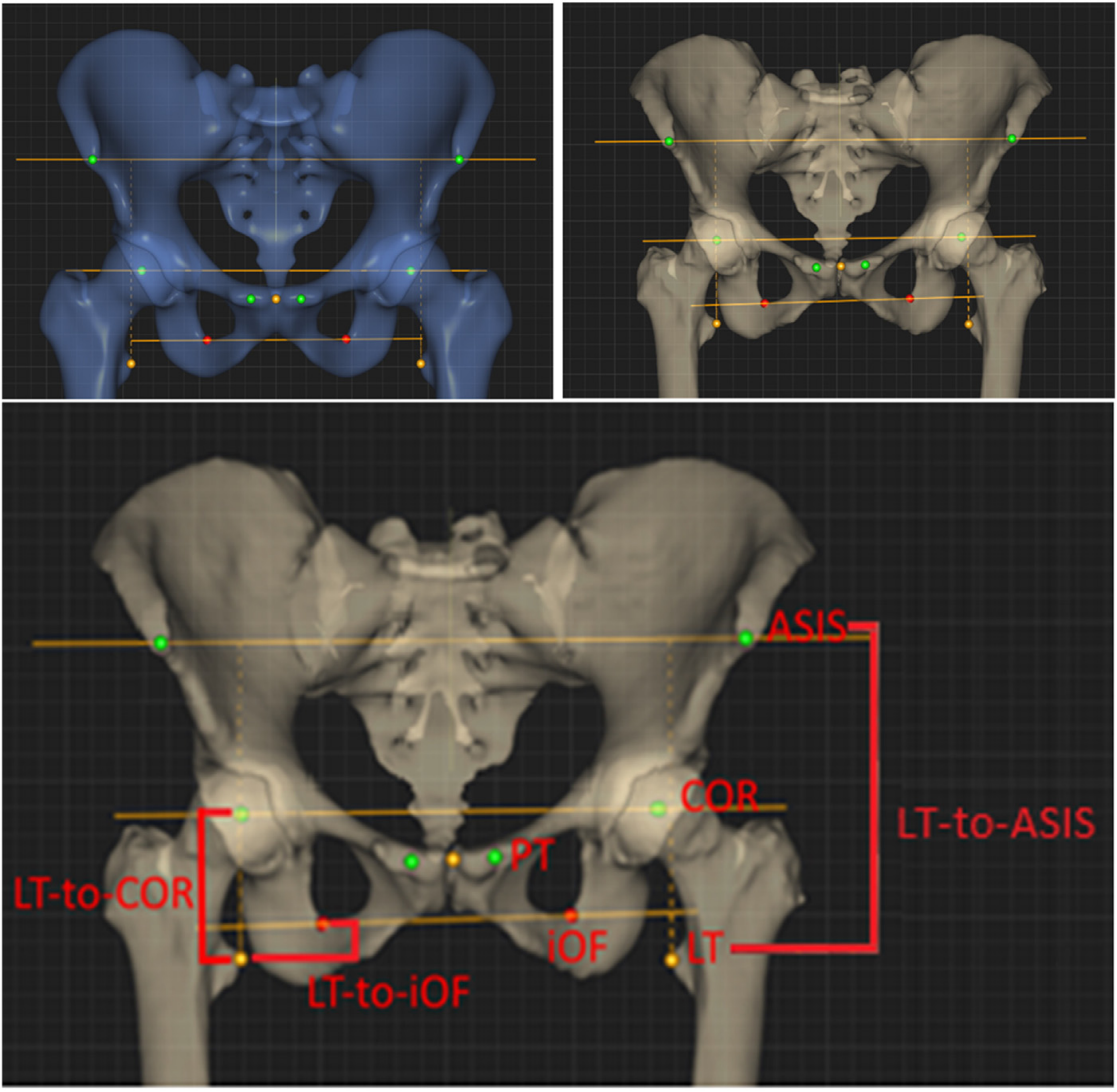
General landmark and measurement selection of the ASIS, COR, and iOF axes for hip length measurement on the corresponding model (top left) and how they are mapped on a specific individual’s bones (top right). The 3 different measures of hip length (LT-to-ASIS, LT-to-iOF, and the control hip length measure, LT-to-COR) (bottom).

The following bony landmarks were mapped on each individual’s bones: L/R pubic tubercles (PT), ASIS, acetabular centers of rotation (COR), inferior obturator foramen (iOF), and lesser trochanters (LT) (Fig. 1). Furthermore, in the 106 scans with the corresponding tibiae, the following additional landmarks were mapped: medial and lateral epicondyles, mid-epicondylar point (MidE), and MM. Only 106 of 234 pelvic scans contained whole-limb data. A list of abbreviations used is available in Table 1.

**Table 1:** List of abbreviations.

Anatomic axes:	
ASIS	Anterior superior iliac spine
COR	Acetabular centers of rotation
iOF	Inferior obturator foramen
LatE	Lateral epicondyles
LT	Lesser trochanters
MedE	Medial epicondyles
MidE	Mid-epicondylar (femoral) point
MM	Medial malleolus
PT	Pubic tubercles
Morphological length measures:	
LT-to-ASIS	Hip length measure A
LT-to-COR	Hip length measure control
LT-to-iOF	Hip length measure B
MidE-to-COR	Femur length
MM-to-MidE + MidE-to-COR	Limb length
Terminology:	
FLD	Femur length discrepancy
HLD	Hip length discrepancy
LLD	Limb length discrepancy
SOMA	Stryker Orthopaedic Modeling Analytics

**Figure 2:**
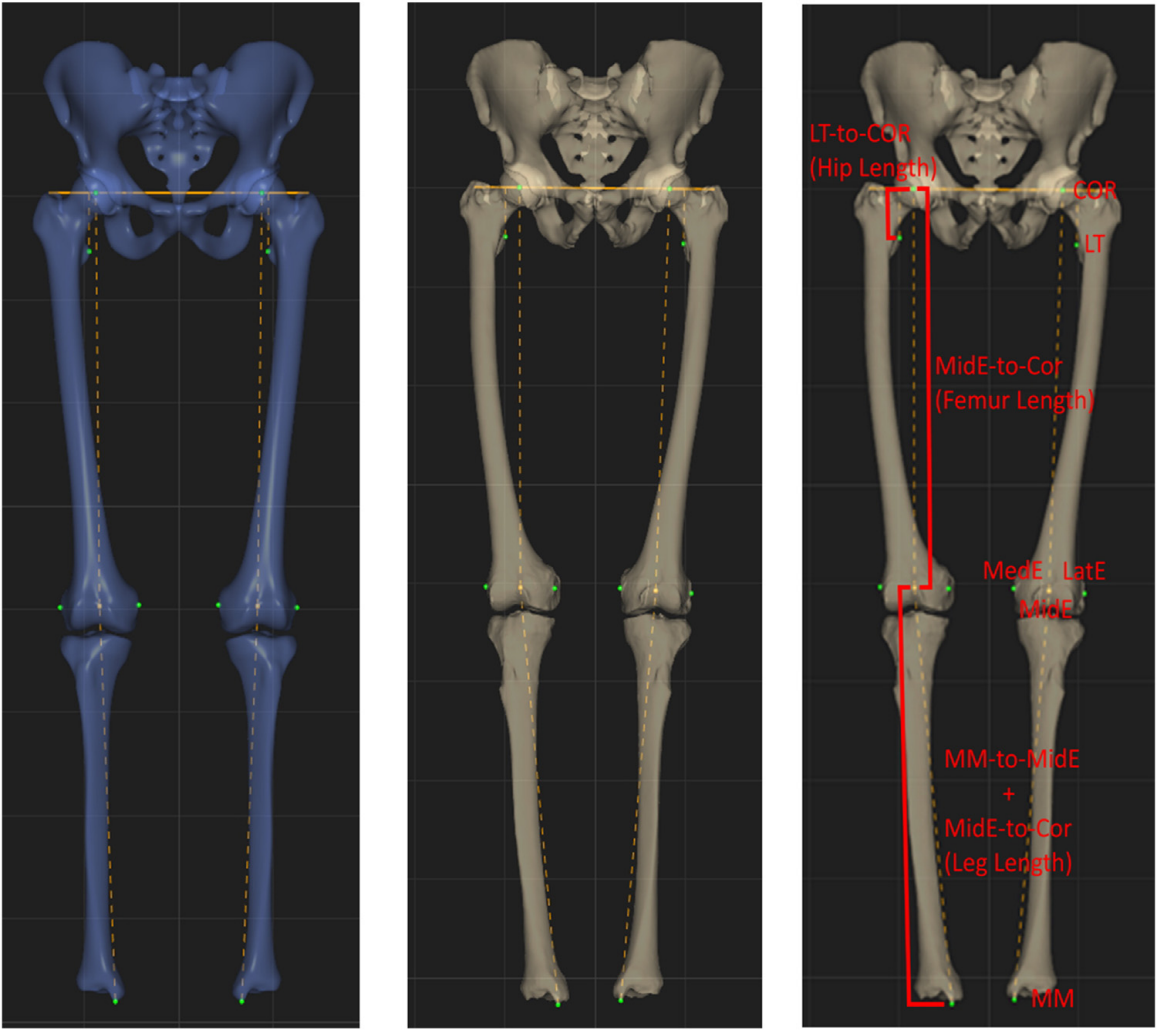
Measurements on the correspondence model used to calculate and compare hip length, femur length, and limb length discrepancies (left). The same measurements were taken on the 106 patients in the subgroup in which the pelvis and corresponding femoral and tibial models were available (example shown in middle). Visual representation of hip length (LT-to-COR), femur length (MidE-to-COR), and limb length (MM-to-MidE + MidE-to-COR) (right).

The average pelvic lateral offset L/R discrepancy due to pelvic asymmetry was calculated as the difference in distance from each side’s COR to a sagittal plane through a mid-PT point. To isolate hip length discrepancies due to pelvic asymmetry, the distances from the LT-to-ASIS axis and LT-to-iOF axis were compared to the LT-to-COR axis (control). In the 106 patient subgroup, hip length (LT-to-COR), femur length (MidE-to-COR), and limb length (MM-to-MidE + MidE-to-COR) L/R discrepancies (hip length discrepancy [HLD], femur length discrepancy [FLD], and limb length discrepancy [LLD]; respectively) were calculated and compared (Fig. 2).

Lastly, the effect of the pelvic reference on measured changes to hip length on the operative side only (ie, preoperative vs postoperative) was examined. A 10 mm increase in hip length perturbation was simulated, and the difference in measured hip length change was calculated using the iOF and ASIS references relative to the COR axis reference.

## Results

Overall, 234 pelvic and their corresponding femora were analyzed (146 males and 88 females), of which 207 were of Caucasian ethnicity, 12 were of Asian ethnicity, and the remaining 15 were of Middle Eastern or African descent. Corresponding tibial scans were also available in 106 of the 234 patients (52 males and 54 females), of whom 87 were of Caucasian descent, 12 were of Asian descent, and 7 were of African ethnicity.

**Figure 3:**
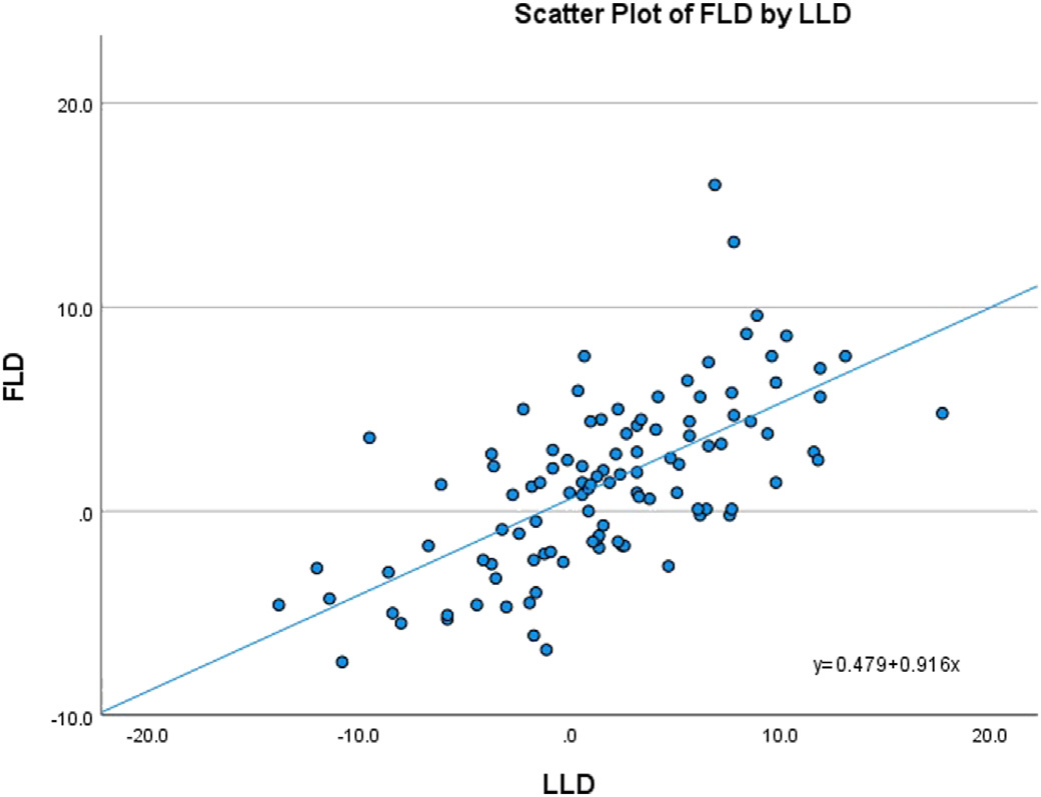
Scatterplot of femur and limb length discrepancies.

**Figure 4:**
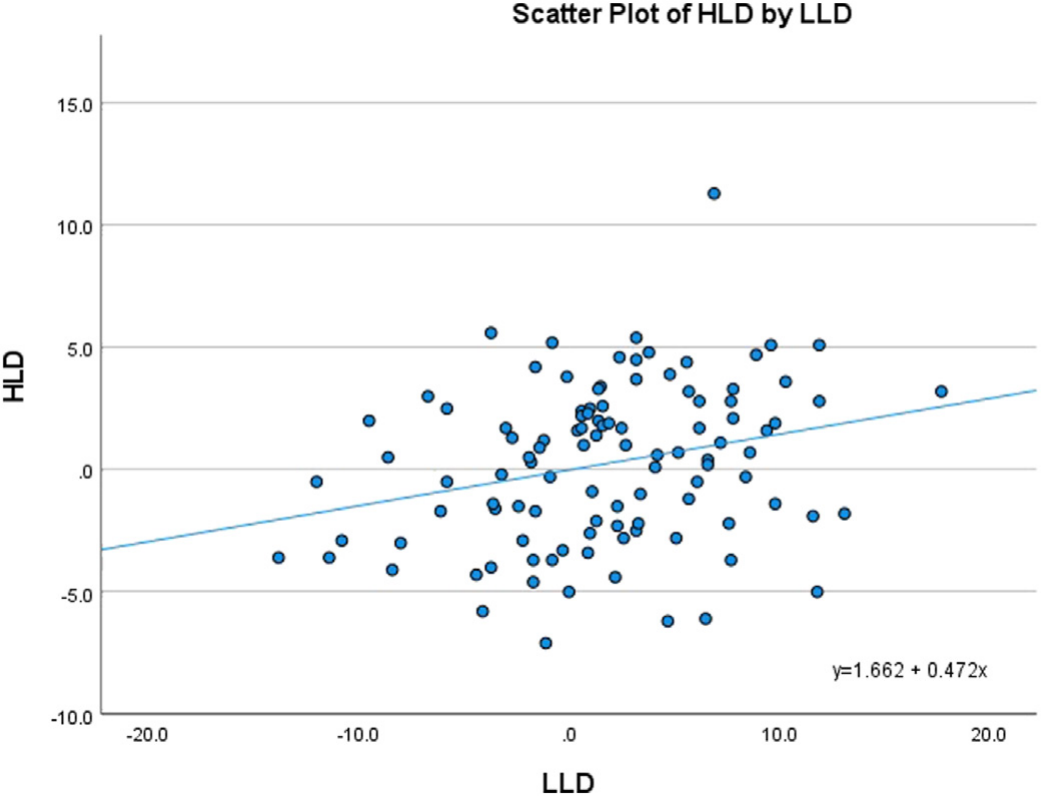
Scatterplot of hip and limb length discrepancies.

Average HLD, FLD, and LLD between L/R sides were 2.7 ± 1.8 mm, 3.5 ± 2.7 mm, and 4.8 ± 3.8 mm, respectively (n = 106). The average discrepancy difference between each individual’s hip length and limb length was 4.8 ± 3.7 mm, while the average difference between FLD and LLD was 3.4 ± 2.8 mm. There was significant correlation between FLD and LLD (r = 0.656, *P* < .001; Fig. 3) and less correlation between HLD and LLD (r = 0.264, *P* = .006; Fig. 4).

**Figure 5:**
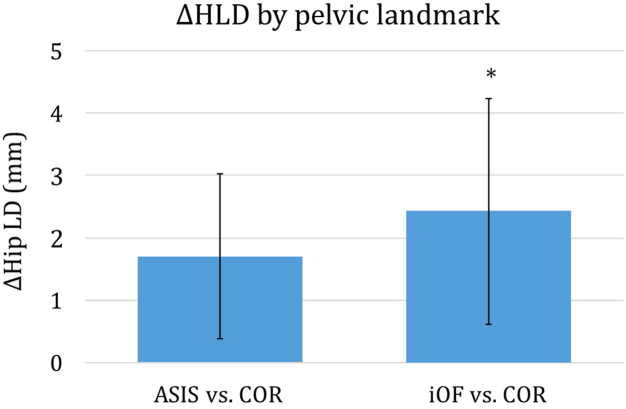
The difference in hip length discrepancy relative to the CORs when using the ASIS or inferior obturator foramen axes as the pelvic reference.

The average difference in COR-lateral offset (COR-to-mid PT plane) between L/R sides was 2.2 ± 1.6 mm (5.4 mm @ 2 SD). The L/R HLD using an LT-to-ASIS measurement differed slightly by 1.7 ± 1.3 mm (*P* = .27) relative to control (LT-to-COR, Fig. 5). Differences in LT-to-iOF L/R discrepancies relative to the COR axis were greater and found to be significant (2.4 ± 1.80 mm, *P* < .05, Fig. 5).

Selecting a different pelvic reference point had little effect on the measured hip length changes due to a simulated perturbation of a 10 mm hip length increase on the operative side (eg, the difference in hip length change using COR relative to ASIS: 0.5 ± 0.6 mm).

## Discussion

This is the largest study to date to evaluate pelvic asymmetry using CT scan in such a diverse population. Using the SOMA software, the relationship between LT-to-COR and other landmarks such as the LT-to-ASIS and LT-to-iOF was evaluated to determine the most accurate reference points for calculating hip length. In addition to evaluating HLDs, FLDs were determined, and this study demonstrated variability relative to overall LLD (average discrepancy difference relative to limb length: 4.8 ± 3.7 mm for hip and 3.4 ± 2.8 mm for femur) with little correlation between the methods.

ASIS vs iOF-based assessment of hip length demonstrated a difference in calculated hip length. While the ASIS axis is more reliable than the iOF axis, measured HLD using an LT-to-ASIS can still vary as much as 4.3 mm (@ 2 SD) relative to a LT-to-COR axis reference. The hip length difference was reported as being higher with iOF than ASIS-based measurements. This difference in hip length was up to 17.0 mm for iOF and 12.5 mm for ASIS measurements. Traditionally, the nonpathologic opposite side would be considered a normal reference point when using CT scans for preoperative planning for implant placement. However, this may overlook pre-existing pelvic asymmetry if only limited pelvic and hip CTs are used that don’t extend to the entire lower limb. This could potentially lead to the navigation system inappropriately calculating a HLD of up to 4.5 mm in some cases prior to implantation of any prosthesis.

While landmark selection can greatly affect calculated L/R preoperative discrepancy, this study demonstrates that the exact landmark reference selected by these CT-based imaging systems (LT-to-ASIS vs LT-to-iOF vs LT-to-COR) has little effect on calculated changes in hip length of the operative side when a perturbation in hip length is performed to simulate the insertion of a prosthesis on the contralateral side. It can be concluded that for contralateral limb length changes intraoperatively, all measured points did not differ significantly when measuring a simulated hip length perturbation.

This study population showed widespread pelvic asymmetry, and the anatomical landmarks of the opposite side cannot be relied upon for predicting the anatomy of the affected side. With regards to pelvic asymmetry, the average difference in COR lateral offset (COR-to-mid PT plane) between sides was 2.2 ± 1.6 mm (5.4 mm @ 2 SD). The average limb-length discrepancy of 4.8 mm in this study is in accordance with the study by Knutson et al. [25], which demonstrated a 5 mm discrepancy in 90% of the general population. Although the proportion of patients with a large amount of asymmetry (>5 mm) was not great and similar to previous reports [9], the variability of certain reference points makes navigation selection important due to the effect on limb length and offset calculations.

Achieving the correct limb length after total hip arthroplasty (THA) is crucial in achieving a good functional result. LLD after THA will result in patient dissatisfaction and increased complications [26]. Fujimaki et al. [27] reported that a LLD >0.5 cm following THA resulted in worse clinical outcome scores compared with patients with discrepancies <0.5 cm postoperatively. LLDs of more than 1-2 cm have been shown to cause gait abnormalities, which lead to increased energy expenditure. This can be critical for elderly patients with substantial pulmonary, cardiac, or neuromuscular disease [28]. LLD is also shown to increase the risk of aseptic loosening, implant failure and pelvic obliquity [29].

Computer-assisted surgery (CAS) in THA has been shown to improve the accuracy and reproducibility of implant positioning, reducing the outliers [30]. However, identifying the outliers with pelvic asymmetry prior to templating for navigation is crucial for accurate placement of the implant in THA. This study population had widespread pelvic asymmetry, which has implications for correct implant placement depending on which pelvic landmarks the surgeon uses as reference points. With better understanding of pelvic asymmetry trends, evolving technology, and utilization of multiple anatomical landmarks, computer-assisted THA may enable surgeons to be more accurate in their component positioning.

Recreation of symmetrical limb length and offset with successful THA prevents complications such as low back pain, impaired abductor function, and dislocation [31]. Restoration of lateral offset is an important step in recreating the abductor complex’s function. If this is not adequately recreated, it can result in pelvic obliquity which leads to perceived or functional LLD. This results in increasing pain in the trochanteric region and patient dissatisfaction. The average difference in lateral offset of 2.2 ± 1.6 mm between sides in this study demonstrates the difficulties in accurately restoring lateral offset if the opposite side anatomy is used as a reference. Boulay et al. [17] have reported in an anatomical study that there is a clockwise rotation of the iliac wings while the lower part of pelvis, the pubic symphysis, rotates counterclockwise. This rotational asymmetry of the pelvis might explain the difference in the lateral offset of the hip in this study. Regardless of its cause, it is crucial to identify this difference in the offset between 2 hips before THA.

With the ongoing relentless global improvement in 3D imaging, AI, data mining, and cloud computing, it is not improbable that clinicians in the future will have the capacity to correlate patient-perceived LLD and other patient-reported outcomes with individualized bone morphology indices and landmarks to better preoperatively and intraoperatively plan and then execute the insertion of prostheses using image-based navigation tools. This study will help in the analysis of pelvic bony landmarks and prevent referencing errors in computer-assisted surgery, which will hopefully improve accuracy in achieving ideal postoperative limb length and offset and hopefully result in improved patient satisfaction. Until CAS software based on analysis of deformity, pelvic asymmetry, and its effect on implant orientation are available, clinicians should be aware that reliance on the anatomical landmarks from the opposite hip may impair optimal implant position and lead to erroneous implant placements. Although HLD is significantly correlated to LLD, the correlation is not strong. As such, a broader awareness of the entire limb morphology may be necessary to accurately navigate CT based THR to avoid erroneous extrapolation of hip length to limb length when using limited imaging. In the absence of CAS calibrated with full-length CTs that make use of accurate landmarks and account for any present pelvic asymmetry, traditional templating, long limb films, traditional intraoperative tests of limb length, and awareness of existing deformity still hold value in optimal implant positioning even when employing existing CT-based CAS and robotic technologies.

There are some limitations to this study that should be recognized. This study utilized a CT database to evaluate pelvic morphology, and no clinical correlation was attempted. Even though this study has a mix of most of the ethnic population, the predominant population is Caucasian. This can potentially skew the data for generalizability to other ethnic groups with regards to pelvic asymmetry. Additionally, tibial scans were included in only 106 of the 234 cases, which reduces the power of our commentary on limb length interpretation. There is also a gender disparity with fewer females represented in the cohort than males.

## Conclusions

Pelvic asymmetry was common in this study. The LT-to-COR was demonstrably the most reliable measure of HLD due to pelvic asymmetry although a simulated change in hip length could be reliably measured using any of the studied bony landmarks. The implications of these findings are that while current CT-based robotic THR software systems already give measurements of global offset and hip length, these measurements do not consider pelvic asymmetry. Operating surgeons are therefore not given confidence ranges to represent the potential impact of asymmetry on global offset and hip length. Instead, surgeons are currently only presented with absolute numbers that may incur implant placement error should significant pelvic asymmetry be present in a given patient.

## Funding

Funding for the Article Processing Fee (APC) was kindly provided by The Prince Charles Hospital, Chermside, Brisbane, Queensland, Australia.

## Conflicts of interest

R. Crawford receives royalties from Stryker Corporation in relation to products not investigated in this paper, receives research support from Stryker Orthopaedics for research projects of which he is the principal investigator, and is on the editorial board/governing board for the Journal of Arthroplasty. T. Wuestemann is a paid employee and has stock options for Stryker. M. Thompson is a paid employee and receives stock options from Stryker. S. Whitehouse’s position is partially supported via external institution by Stryker Australia and Stryker EU; the other author declares no potential conflicts of interest.

For full disclosure statements refer to https://doi.org/10.1016/j.artd.2023.101206.

## Appendix A Supplementary data

Conflict of Interest Statement for Crawford

Conflict of Interest Statement for Wuestemann

Conflict of Interest Statement for O’Callaghan

Conflict of Interest Statement for Thompson

Conflict of Interest Statement for Whitehouse
