# Supplementary material for: Three-Dimensional CT-Based Limb Length Evaluation Is Highly Dependent on Anatomical Landmark Selection and Pelvic Asymmetry
Source: Arthroplast Today. 2023 Sep 18;23:101206. doi: 10.1016/j.artd.2023.101206 (PMC10514420; doi:10.1016/j.artd.2023.101206)
Supplement: Conflict of Interest Statement for Crawford [file mmc1.pdf]

INDIVIDUAL CONFLICT OF INTEREST STATEMENT

American Association of Hip and Knee Surgeons

(Adopted from the American Academy of Orthopaedic Surgeons disclosure statement)

The following form **must be filled out completely and submitted by each author (example, 6 authors, 6 forms). All items require a response. If there is no relevant disclosure for a given item, enter "*None.*"**

Manuscript Title: Three-Dimensional CT-Based Leg Length Evaluation is Highly Dependent on Anatomical Landmark Selection and Pelvic Symmetry

1. Royalties from a company or supplier (The following conflicts were disclosed)

Royalties from Stryker Corporation in relation to products not investigated in this paper

2. Speakers bureau/paid presentations for a company or supplier (The following conflicts were disclosed)

None

3A. Paid employee for a company or supplier (The following conflicts were disclosed)

None

3B. Paid consultant for a company or supplier (The following conflicts were disclosed)

None

3C. Unpaid consultants for a company or supplier (The following conflicts were disclosed)

None

4. Stock or stock options in a company or supplier (The following conflicts were disclosed)

None

5. Research support from a company or supplier as a Principal Investigator (The following conflicts were disclosed)

Stryker Orthopaedics support research projects of which I am Principal Investigator

6. Other financial or material support from a company or supplier (The following conflicts were disclosed)

None

7. Royalties, financial or material support from publishers (The following conflicts were disclosed)

None

8. Medical/Orthopaedic publications editorial/governing board (The following conflicts were disclosed)

Editorial board for the Journal of Arthroplasty

9. Board member/committee appointments for a society (The following conflicts were disclosed)

None

Each author must sign AND print or type his/her name, date and submit a separate form

In addition, one BLINDED Conflict of Interest form (no author names used) should be submitted per manuscript with all author disclosures.

RW Crawford	Ross Crawford	10 March 2022
Author Name (Print or Type)	Author Signature	Date
