# Supplementary material for: Three-Dimensional CT-Based Limb Length Evaluation Is Highly Dependent on Anatomical Landmark Selection and Pelvic Asymmetry
Source: Arthroplast Today. 2023 Sep 18;23:101206. doi: 10.1016/j.artd.2023.101206 (PMC10514420; doi:10.1016/j.artd.2023.101206)
Supplement: Conflict of Interest Statement for Whitehouse [file mmc5.pdf]

CONFLICT OF INTEREST STATEMENT

The Journal of Arthroplasty

(Adopted from the American Academy of Orthopaedic Surgeons disclosure statement)

The following form **must be filled out completely and submitted by each author (example, 6 authors, 6 forms)**. **If no discloser is required, please write/type "none" at the end of each sentence.**

Manuscript Title: Three-Dimensional CT-Based Leg Length Evaluation is Highly Dependent on Anatomical Landmark Selection and Pelvic Symmetry

1. Royalties from a company or supplier (The following conflicts were disclosed)

None

2. Speakers bureau/paid presentations for a company or supplier (The following conflicts were disclosed)

None

3A. Paid employee for a company or supplier (The following conflicts were disclosed)

None

3B. Paid consultant for a company or supplier (The following conflicts were disclosed)

None

3C. Unpaid consultants for a company or supplier (The following conflicts were disclosed)

None

4. Stock or stock options in a company or supplier (The following conflicts were disclosed)

None

5. Research support from a company or supplier as a Principal Investigator (The following conflicts were disclosed)

Position partially supported via external institution by Stryker Australia and Stryker EU

6. Other financial or material support from a company or supplier (The following conflicts were disclosed)

None

7. Royalties, financial or material support from publishers (The following conflicts were disclosed)

None

8. Medical/Orthopaedic publications editorial/governing board (The following conflicts were disclosed)

None

9. Board member/committee appointments for a society (The following conflicts were disclosed)

None

Each author must sign AND print or type his/her name, date and submit a separate form

In addition, one BLINDED Conflict of Interest form (no author names used) should be submitted per manuscript with all author disclosures.

Sarah L Whitehouse	SL Whitehouse	10 March 22
Author Name (Print or Type)	Author Signature	Date
